# Effect of financial support on reducing the incidence of catastrophic costs among tuberculosis-affected households in Indonesia: eight simulated scenarios

**DOI:** 10.1186/s40249-019-0519-7

**Published:** 2019-02-02

**Authors:** Ahmad Fuady, Tanja A. J. Houweling, Muchtaruddin Mansyur, Erlina Burhan, Jan Hendrik Richardus

**Affiliations:** 1 Department of Public Health, Erasmus MC, University Medical Centre Rotterdam, P.O. Box 2040, 3000CA, Rotterdam, the Netherlands; 2 Department of Community Medicine, Faculty of Medicine, Universitas Indonesia, Jakarta, Indonesia; 3 Department of Respiratory and Pulmonology, Persahabatan Hospital – Faculty of Medicine, Universitas Indonesia, Jakarta, Indonesia

**Keywords:** Tuberculosis, Multidrug-resistant tuberculosis, Catastrophic cost, Social protection, Financial support, Cash transfer, Indonesia

## Abstract

**Background:**

The World Health Organization’s End Tuberculosis Strategy states that no tuberculosis (TB)-affected households should endure catastrophic costs due to TB. To achieve this target, it is essential to provide adequate social protection. As only a few studies in many countries have evaluated social-protection programs to determine whether the target is being reached, we assessed the effect of financial support on reducing the incidence of catastrophic costs due to TB in Indonesia.

**Methods:**

From July to September 2016, we interviewed adult patients receiving treatment for TB in 19 primary health centres in urban, sub-urban and rural area of Indonesia, and those receiving multidrug-resistant (MDR) TB treatment in an Indonesian national referral hospital. Based on the needs assessment, we developed eight scenarios for financial support. We assessed the effect of each simulated scenario by measuring reductions in the incidence of catastrophic costs.

**Results:**

We analysed data of 282 TB and 64 MDR-TB patients. The incidences of catastrophic costs in affected households were 36 and 83%, respectively. Patients’ primary needs for social protection were financial support to cover costs related to income loss, transportation, and food supplements. The optimum scenario, in which financial support would be provided for these three items, would reduce the respective incidences of catastrophic costs in TB and MDR-TB-affected households to 11 and 23%. The patients experiencing catastrophic costs in this scenario would, however, have to pay high remaining costs (median of USD 910; [interquartile range (IQR) 662] in the TB group, and USD 2613; [IQR 3442] in the MDR-TB group).

**Conclusions:**

Indonesia’s current level of social protection is not sufficient to mitigate the socioeconomic impact of TB. Financial support for income loss, transportation costs, and food-supplement costs will substantially reduce the incidence of catastrophic costs, but financial support alone will not be sufficient to achieve the target of 0% TB-affected households facing catastrophic costs. This would require innovative social-protection policies and higher levels of domestic and external funding.

**Electronic supplementary material:**

The online version of this article (10.1186/s40249-019-0519-7) contains supplementary material, which is available to authorized users.

## Multilingual abstracts

Please see Additional file 1 for translations of the abstract into the six official working languages of the United Nations.

## Background

Poverty is closely related to tuberculosis (TB), both as a risk and as an effect. People in low-income households not only have a higher risk of TB infection, but once they are infected, the high costs associated with diagnosis and treatment may reduce them to poverty [1, 2]. Although almost all countries provide drugs free of charge to patients with susceptible TB and multidrug-resistant TB (MDR-TB), TB patients still face high direct non-medical costs such as those for travel, food, and nutritional supplements [3–5]. Indonesia has the world’s third largest number of estimated TB cases with the incidence of 842 000 (95% Confidence Interval [*CI*] 767 000–919 000) cases. Despite its free TB services, our recent study revealed that the costs incurred during the treatment phase constituted more than three-quarters of the total costs [6]. These high costs can negatively affect treatment adherence, clinical outcomes, and drop-out rates, thereby further increasing future costs. The high costs also carry the risk of plunging TB patients and their families into poverty, or into even deeper poverty for those already living in poverty [7–11].

In response to this socioeconomic burden, the World Health Organization’s (WHO’s) End TB Strategy aims by 2020 to reduce to zero the percentage of TB-affected families that face catastrophic costs. Catastrophic costs due to TB are defined as the total costs – i.e., all direct and indirect costs, including income loss — that exceed a specific threshold (e.g., 20%) of a household’s annual income [12]. One obvious option for attaining this target is by providing adequate social protection. In 2014, the Indonesian government started a national health insurance scheme that covers all the medical costs – including those of TB treatment – incurred in primary, secondary and tertiary healthcare. This scheme has substantially reduced direct medical costs. However, direct medical costs are not the only costs patients face in the trajectory from the pre-diagnostic phase to treatment completion [4, 13]. In 2016, the previous study we conducted in Indonesia showed that total costs consisted largely of direct non-medical costs and income loss [6]. As these are not covered by the health insurance scheme, TB patients are still at a high risk of facing catastrophic costs. This highlights the importance of providing additional financial protection to cover direct non-medical costs and income loss [13].

There are three approaches to delivering additional social protection [14]. The first is the TB-specific approach, which offers protection only to TB patients or TB-affected households, for example by providing food-supplements or travel vouchers to those undergoing TB treatment in health facilities that are linked to the network of the National Tuberculosis Program (NTP) [14, 15]. The second, the TB-inclusive approach, is a broader intervention in which TB patients or TB-affected households are one of the inclusion criteria in a social-protection program. The third, the TB-inclusive approach, involves protection policies that do not explicitly include TB patients or TB-affected families in their eligibility criteria but include TB risk-reduction strategies for groups at a high risk for TB infection, such as general cash transfers and premium-free national health insurance for people in poor households.

However, there is little evidence on the effectiveness of financial protection (including cash transfers) in reducing the incidence of catastrophic costs due to TB, particularly in TB high-burden countries (HBCs). Although recent studies have shown that cash transfers could defray the costs endured by TB-affected households [15, 16], the transfers in question were either given conditionally (on the basis of adherence to the intervention program), or were given on the basis of published national average cost data rather than of patients’ actual needs. There is limited evidence on the effect of social-protection schemes that take account of patients’ needs and preferences [17].

In this study we therefore aimed to measure the socioeconomic impact of TB and MDR-TB (including the incidence of catastrophic costs), and to assess patients’ perceived needs for social protection in Indonesia. Additionally, to assess the effects of financial support on the incidence of catastrophic costs due to TB and MDR-TB in poor and non-poor households, we developed and ran hypothetical scenarios in which patients were offered different combinations of financial support.

## Methods

### Study design

To measure the socioeconomic impacts of TB and the perceived needs for social protection, we interviewed TB and MDR-TB patients. For TB patients, we applied stratified clustered sampling in three districts of Java, one representing an urban area of Indonesia (Jakarta), one representing a suburban area (Depok), and one representing a rural area (Tasikmalaya). In each district, we randomly selected 5–8 primary health centres (PHCs) that were linked with the Indonesian NTP. Then, from July to September 2016, we selected consecutive TB patients consecutive TB patients as they registered at these PHCs. Assuming that the incidences of TB-affected household facing catastrophic costs were 20% (urban), 25% (suburban) and 30% (rural), assuming a 1:1:1 ratio of TB incidence in urban, suburban and rural district, and assuming a power of 0.80, we required a minimum of 90 TB patients who met the inclusion criteria in each district. We included adults aged 18 years or above who had been diagnosed with TB and had either received TB treatment for at least one month or had finished the treatment no more than one month previously. In this study, we focused on pulmonary TB and excluded extra-pulmonary TB patients because of potentially different seeking care pattern and costs. With regard to MDR-TB, we interviewed adult patients who had been diagnosed on the basis of GeneXpert® (Cepheid, Sunnyvale, CA, USA) or sputum culture and had been undergoing treatment for at least one month in Persahabatan Hospital, an MDR-TB referral hospital in Jakarta.

In both groups of patients, we assessed the following variables: the incidence of catastrophic costs, the socioeconomic impacts of TB or MDR-TB, and patients’ perceived needs for social protection. On the basis of the needs assessment, we then developed several scenarios for financial support. In each scenario, we measured the reduction in the incidence of catastrophic costs after the hypothetical provision of financial support.

### Socioeconomic impacts due to TB and MDR-TB

To measure the socioeconomic impacts of TB, we used the Tool to Estimate Patient Costs [12, 18], which we adapted to the Indonesian context, also translating it to Indonesian Bahasa. We recruited ten medical students and public health graduates as interviewers and trained them in the use of the adapted tool. We studied the incidence of TB-affected households facing catastrophic costs; patients’ perception of the their TB or MDR-TB is having on their households’ financial capacity expressed on a scale of 1–5, from no problem to a very serious problem; coping strategy (loaning money or selling property); job and income loss due to TB; and the proportional reduction in patients’ and households’ income. Patient and household income loss were calculated both in absolute terms (in United States dollars, USD) and in relative terms (percentage of loss of previous income).

As well as collecting information on all types of cost (i.e., direct medical costs, direct non-medical costs, and indirect costs) that had been incurred by the TB-affected households in the period between the pre-diagnostic phase and treatment completion, we also collected information on these households’ annual income. Following the latest WHO protocol, we measured the incidence of catastrophic costs (defined as total direct and indirect costs) that exceeded 20% of each TB-affected household’s annual income [6, 12]. Details of the methods we used to calculate the incidence of catastrophic costs due to TB are provided in our previous study [6].

### Perceived needs for social protection

To assess patients’ knowledge of social protection and their perceived needs for additional social protection, we added three questions at the end of the adapted tool: “Have you ever heard of social protection?”, followed by an open question: “If yes, what is social protection? Can you explain it?”

Patients’ answers were grouped according to six types of social protection: general government aid for the poor; government aid for healthcare; direct government aid (general cash transfer); government aid for education: government aid for transportation costs; and other government aid. A patient’s inability to name or explain any type of social protection was defined as ‘did not know’. These questions were important to our ability to assess patients’ knowledge before questioning them on their needs for social protection. After obtaining the patients’ answers, the interviewers explained the definition of social protection and gave examples of several types of social-protection scheme [19]. They then asked the patients whether they needed any social protection, or additional protection if they already receiving.

Patients who stated that they needed social protection were asked to choose one cost item they wanted to be covered, and its value in Indonesian Rupiahs (IDR). These items comprised consultation fee per visit, transportation costs per visit, food costs per visit, drug costs per month, income loss per month, and food-supplement costs per month. Food-supplement costs were defined as a patient’s monthly spending on nutritional or food supplements such as vitamins, fruits, milk, meats, or other nutritional supplements that were consumed either with or without a doctor’s TB-related recommendation [19]. After the patients’ answers had been obtained, the cost items that needed to be covered were listed in order of priority (from those that had been indicated most to those that had been indicated least). The median (interquartile range [IQR]) of these cost items was then calculated. In the scenarios that we developed, we then used the median values of these cost items as the value of financial support.

### Effects of financial support

On the basis of the needs assessment, we selected the three cost items that patients chose, and then developed several hypothetical scenarios for financial support. These comprised the following: no provision of a cash transfer (baseline); the provision of a cash transfer to cover a single cost item (i.e., income loss, transportation costs, or food-supplementation); and the provision of a cash transfer to cover a combination of two or three cost items. In total, we developed eight such scenarios. As well as the baseline scenario (no cash transfers; Scenario I), we formulated seven hypothetical cash-transfer scenarios for the following: (II) transportation costs for all patients, (III) food-supplement costs for all patients, (IV) income loss for patients who had lost their jobs, (V) income loss for patients who lost their income whether or not they had lost their jobs, (VI) a combination of transportation costs and income loss, (VII) a combination of food-supplement costs and income loss, and (VIII) a combination of transportation costs, food-supplement costs, and income loss.

We simulated the hypothetical scenarios in the people who had been included in this study, assuming that the cash transfers had been made after patients had started TB or MDR-TB treatment. The value of the cash transfer (*CT*) for specific cost items was extrapolated to a complete treatment period (denoted $$ CT\ {\displaystyle \begin{array}{c} CI,h\\ {}i\end{array}} $$ in which *CI* identifies a specific cost item, *i* identifies the patient, and *h* identifies his/her household). For transportation costs, the total cash transfer was calculated by multiplying *CT* by the number of PHC or hospital visits during the intensive phase $$ V\ {\displaystyle \begin{array}{c} IP\\ {}i\end{array}} $$ and continuation phase $$ V\ {\displaystyle \begin{array}{c} CP\\ {}i\end{array}} $$ until the expected end date of treatment. For income loss and food-supplement costs, the total value of the cash transfer was calculated by multiplying *CT* by the duration (in months) of the patients’ complete treatment, *M*.

**Box 1 Cash-transfers formula**

**Table:** 

$Total\ CT\ transportation=\sum \limits_{i=1}^n\left(\left({CT}_i^{CI,h}x\ {V}_i^{IP}\right)+\left({CT}_i^{CI,h}x\ {V}_i^{CP}\right)\right)$ $Total\ CT\ income\ loss=\sum \limits_{i=1}^n\left({CT}_i^{CI,h}x\ M\right)$ $Total\ CT\ food\ supplement={\sum}_{i=1}^n\left({CT}_i^{CI,h}x\ M\right)$	

Total costs were defined as the sum of all types of cost, including out-of-pocket payments (*OOPs*) for medical diagnosis and treatment (*OOPM*); OOPs for non-medical expenditures (*OOPNM*); and patients’ and guardians’ income losses (*IN*). After calculating the total simulated costs after the cash transfer (total costs for TB-related services minus the total cash transfer), we estimated the incidence of catastrophic costs after the cash transfer in each scenario. To define catastrophic costs, we used the threshold of 20% of annual household income (denoted $$ \tau {\displaystyle \begin{array}{c} TB\\ {}\end{array}} $$).

$$ I{\displaystyle \begin{array}{c} TB\\ {} NTP\end{array}}=\frac{1}{n\begin{array}{c} TB\\ {} NTP\end{array}}{\sum}_{i=1}^{n\ \begin{array}{c} TB\\ {} NTP\end{array}}1\left(\frac{\sum \begin{array}{c}n{\_}_i\\ {}j=1\end{array}\left(\left( OOPM\begin{array}{c} TB,h\\ {}j\end{array}+ OOPNM\ \begin{array}{c} TB,h\\ {}j\end{array}+ IN\ \begin{array}{c} TB,h\\ {}j\end{array}\ \right)- Total\  CT\ \right)}{y\begin{array}{c}h\\ {}i\end{array}}>\tau \begin{array}{c} TB\\ {}\end{array}\right) $$

TB patient is denoted as *j* while the household is denoted as *i* [12]. If there is more than one TB patient in one household, costs for all patient within the household will be collected or estimated. Although the hypothetical scenarios were based on the optimistic assumption that all patients would receive 100% of the potential cash transfer, some intervention studies have shown that 10–36% of targeted beneficiaries did not receive complete financial support [15, 20, 21]. To obtain valid estimates of the effect of the cash transfers, we ran sensitivity analyses that assumed patients would receive 60, 70, 80, and 90% of the potential cash transfer.

### Data analysis

Data were entered into EpiInfo™ for Windows (Centers for Disease Control and Prevention (CDC), Atlanta, GA, USA) and Microsoft® Office Excel 2010 (Microsoft). For data cleaning and analysis, we used IBM SPSS Statistics for Windows, Version 21 (IBM Corp., Armonk, NY, USA). Categorical variables were displayed as numbers (*n*) and proportions (%). All costs, incomes, and values of financial support for each cost item were collected in IDR and then converted to US Dollars (USD) using the average exchange rate by the World Bank for 2016 (USD 1 = IDR 13389.41) [22]. These numerical data were abnormally distributed and therefore displayed as median values (IQRs).

We compared the socioeconomic impacts, the perceived needs for social protection, and the effect of financial support between poor and non-poor households. A poor household was defined as a household earning below USD 1.9 per capita per day [23]. To compare the socioeconomic impacts due to TB and the effects of financial support between poor and non-poor TB-affected households, we applied generalized linear mixed models with random effects to adjust for a cluster sampling design (19 PHCs). For the MDR-TB group, we used chi-square, Fisher, and Mann-Whitney tests to analyse the impacts between poor and non-poor households. To compare the effects of financial support between scenarios, we used McNemar tests with stratification for cluster sampling for TB (19 PHCs), and without stratification for MDR-TB. For each scenario we used bootstrapping for internal validations of the incidence of catastrophic costs after cash transfer and the average budget per patient required in each scenario (*N* = 1000). The difference was considered statistically significant if *P*-value was below than 0.05.

### Ethical statement

Ethical clearances for this study were obtained from the Ethical Committee at the Faculty of Medicine of Universitas Indonesia–Cipto Mangunkusumo Hospital, Jakarta Indonesia (No. 416/UN2.F1/ETIK/VI/2016) and the Ethical Committee at Persahabatan Hospital, Jakarta, Indonesia (No. DL.01.03/II.3/3817/2016). We provided written and oral explanations to patients before their decision to sign the informed-consent form.

## Results

In total, we analysed the data for 282 TB and 64 MDR-TB patients. The details of patients’ characteristics are provided in our previous study on catastrophic costs due to TB [6].

### Socioeconomic impacts of TB or MDR-TB

The incidence of catastrophic costs due to TB was high, and was significantly higher among MDR-TB-affected households (83%) than among TB-affected ones (36%, *P* <  0.001). Most MDR-TB patients (78%) perceived that TB created moderate to severe problems for the financial capacity of their household. This proportion was lower among TB patients (48%; *P-value* for the difference between MDR-TB and TB patients = 0.009). These financial problems led more MDR-TB patients than TB patients to loan money (50% vs 32%, *P* = 0.042) and to sell property (28% vs 12%, *P* = 0.008) (see Table 1).

**Table 1:** Socioeconomic impact on patients due to TB

Socioeconomic impacts		TB				MDR-TB				P**
		Total (%)	Poor (%)	Non Poor (%)	P*	Total (%)	Poor (%)	Non Poor (%)	P*	
		*n =* 282	*n =* 175	*n =* 107		*n =* 64	*n =* 23	*n =* 41		
Household experiencing catastrophic costs		102 (36)	75 (43)	27 (25)	0.006	53 (83)	19 (83)	34 (83)	1.000	< 0.001
Perceived impact on financial capacity^a^										
No problem		96 (34)	51 (29)	45 (42)		5 (8)	0 (0)	5 (12)		
Slight problem		50 (18)	29 (17)	21 (20)		9 (14)	3 (13)	6 (15)		
Moderate problem		58 (21)	42 (24)	16 (15)		13 (20)	5 (22)	8 (20)		
Serious problem		48 (17)	32 (18)	16 (15)		20 (31)	7 (30)	13 (32)		
	Severe problem	30 (11)	21 (12)	9 (8)		17 (27)	8 (35)	9 (22)		
Perceived impact on financial capacity^b^										
No problem to slight problem		146 (52)	80 (46)	66 (62)	0.030	14 (22)	3 (13)	11 (27)	0.201	0.009
Moderate to severe problem		136 (48)	95 (54)	41 (38)		50 (78)	20 (87)	30 (73)		
Coping strategy										
Loaning money		91 (32)	67 (38)	24 (22)	0.014	32 (50)	14 (61)	18 (44)	0.193	0.042
Selling property		33 (12)	27 (15)	6 (6)	0.029	18 (28)	5 (22)	13 (32)	0.395	0.008
Having an income-earning job		201 (71)	119 (68)	82 (77)	0.139	49 (77)	17 (74)	32 (78)	0.708	0.477
		*n =* 201	*n =* 119	*n =* 82		*n =* 201	*n =* 119	*n =* 82		
Impact on job and income										
Job loss		64 (32)	38 (32)	26 (32)	0.801	34 (69)	10 (59)	24 (75)	0.242	0.001
Sick leave		6 (3)	4 (3)	2 (2)	1.000	2 (4)	1 (6)	1 (3)	1.000	0.657
Income loss		122 (61)	78 (66)	44 (54)	0.117	42 (86)	12 (71)	30 (94)	0.041	0.011
% reduction in median (IQR) of patient’s previous income, %^c^		40 (100)	50 (0)	18 (100)	0.197	100 (54)	100 (100)	100 (0)	0.091	0.002
% reduction in median (IQR) of household’s previous income, %^c^		20 (55)	29 (67)	8 (44)	0.250	40 (38)	27 (72)	42 (27)	0.924	0.299

^a^Perceived impacts in five categories: no problem, slight problem, moderate problem, serious problem, and severe problem, ^b^ Perceived impacts in combined categories: no problem to slight problem and moderate to severe problem, ^c^ Calculated for patients reporting income loss. * *P*-values indicate the statistical significance of differences between poor and non-poor households. ** *P*-values indicate the statistical significance of differences between TB and MDR-TB groups

Among TB-affected households, poor households suffered much more than non-poor households: they had a higher incidence of catastrophic costs (43% vs 25%, *P* = 0.006) and a higher proportion of patients who loaned money (38% vs 22%, *P* = 0.014) and sold property (15% vs 6%, *P* = 0.029). A more substantial proportion also suffered from moderate to severe financial problems (54% vs 38%, *P* = 0.030). Conversely, in MDR-TB-affected households, these economic impacts did not differ significantly between poor and non-poor households.

Before diagnosis, more than three-quarters of all patients had an income-earning job. Among them, TB and MDR-TB caused a high rate of job loss, which was higher in MDR-TB patients than in TB patients (69% vs 32%, *P* = 0.001). In addition to job loss, TB also caused income loss: although some patients maintained their jobs after diagnosis, their income decreased. The proportion of patients who lost income was much higher than the proportion who experienced job loss, and was higher among MDR-TB patients than among TB patients (86% vs 61%, *P* = 0.011).

The extent of income loss among patients who had had an income-earning job before diagnosis was substantial in both relative and absolute terms. Relative income loss was very high among MDR-TB patients (median of 100% [IQR 54%]) and was significantly higher than among TB patients (*P* = 0.002). Patient’s income loss subsequently reduced household income (median of 40% [IQR 38%] in MDR-TB and 20% [IQR 55%] in TB patients, *P* = 0.299). While the point estimates of relative income loss suggest that the loss was much higher among poor TB patients than among non-poor TB ones, the difference was not significantly different.

**Fig. 1:**
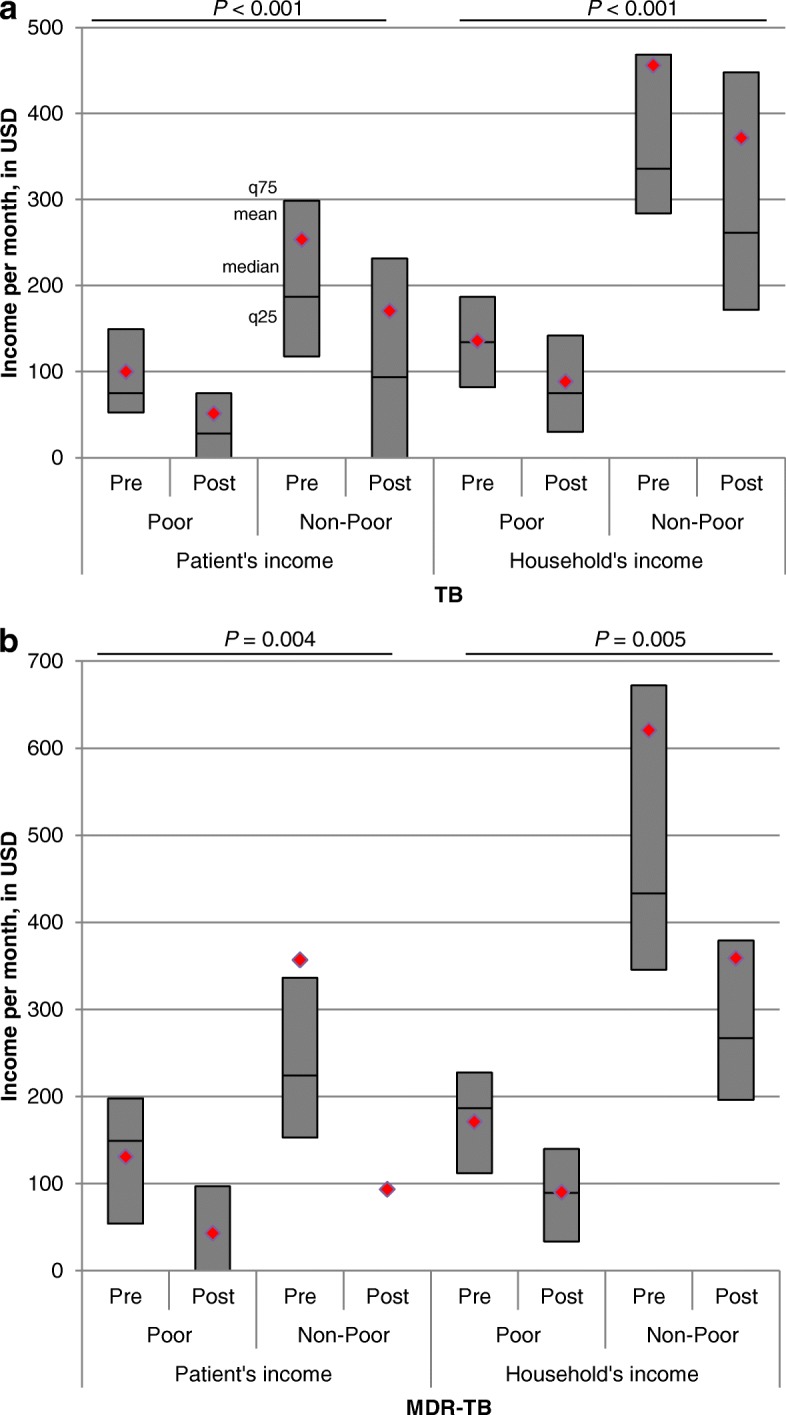
Patient and household income in (**a**) TB and (**b**) MDR-TB-affected households. Pre: income before TB diagnosis. Post: income after TB diagnosis. *P*-values above the bar charts indicates the statistical significance of the absolute difference in income loss between poor and non-poor households. Per bar, red rhombs indicate the mean value of income, upper horizontal lines indicate the q75 value, middle horizontal lines indicate the median value, and lower horizontal lines indicate the q25 value. MDR-TB: Multidrug-resistant tuberculosis; TB: Tuberculosis

Absolute loss (in USD) in patient’s monthly income was higher in non-poor households than in poor households, both in the TB (*P*-value for difference poor and non-poor: < 0.001) and MDR-TB group (*P* = 0.004) (Fig. 1). Household income loss was also greater in non-poor households than in poor households, both in the TB (*P* <  0.001) and MDR-TB group (*P* = 0.005).

### Patients’ perceived needs for social protection

Most patients (84% of TB patients and 80% of MDR-TB patients) did not know existing social-protection schemes (Table 2). Even 81% of patients who had health insurance did not know what social protection was and were unable to name existing social-protection schemes. Knowledge of existing schemes did not differ significantly between TB and MDR-TB patients (*P* = 0.794), or between those with and without health insurance (*P* = 0.112). The forms of social protection that were most commonly named by those who knew of such schemes were government aid for poor people (in general) and government aid for healthcare.

**Table 2:** TB and MDR-TB patients’ knowledge of social-protection schemes

Knowledge of existing social-protection scheme	Type of TB		*P*-value	Having insurance		*P*-value
	TB (%)	MDR-TB (%)		Yes (%)	No (%)	
Did not know what social protection was	236 (84)	51 (80)	0.794	187 (81)	100 (88)	0.112
Knew and could name the following social-protection schemes:	46 (16)	13 (20)		45 (19)	14 (12)	
Government aid for poor people (in general)	19 (7)	4 (6)		19 (8)	4 (4)	
Government aid for healthcare	13 (5)	2 (3)		11 (5)	4 (4)	
Direct government aid, cash transfer^a^	11 (4)	1 (2)		9 (4)	3 (3)	
Government aid for transportation costs	0 (0)	3 (5)		3 (1)	0 (0)	
Government aid for education	1 (0)	2 (3)		2 (1)	1 (1)	
Other government aid	0 (0)	1 (2)		0 (0)	1 (1)	

^a^known as *Bantuan Langsung Tunai* in Indonesian Bahasa

After it had been explained what social protection was, most patients perceived that they needed social protection. The perceived need was higher among MDR-TB patients than among TB patients (95% vs 73%, *P* = 0.004). Perceived need did not differ significantly between poor and non-poor patients in either the TB group (75% vs 70%, *P* = 0.334) or the MDR-TB group (100% vs 93%, *P* = 0.547).

TB and MDR-TB patients all indicated that the three cost items that most needed to be covered were income loss (indicated by 24% of TB patients and 34% of MDR-TB patients); transportation costs (19 and 42%); and costs for food supplements (15 and 8%) (Table 3)

**Table 3:** Perceived needs for financial support for each cost item and their actual costs, median (IQR), in USD

Cost items	Perceived needs^a^							Actual costs^b^		
	Total		Poor		Non-Poor		P**	Total	Poor	Non-Poor
	*n* (%)	Median (IQR)	*n* (%)	Median (IQR)	*n* (%)	Median (IQR)		Median (IQR)	Median (IQR)	Median (IQR)
TB	*n =* 282		*n =* 175		*n =* 107					
Income loss, *per month*	68 (24)	75 (112)	40 (23)	75 (112)	28 (26)	75 (131)	< 0.001	86 (127)^c^	75 (77)^c^	149 (185)^c^
								75 (112)^d^	61 (87)^d^	142 (174)^d^
Transportation, *per visit*	54 (19)	4 (5)	42 (24)	2 (2)	12 (11)	7 (17)	< 0.001	0 (1)	1 (1)	0 (0)
Food-supplement, *per month*	42 (15)	22 (37)	24 (14)	22 (21)	18 (17)	34 (60)	< 0.001	2 (11)	1 (9)	3 (15)
Consultation, *per visit*	11 (4)	13 (19)	6 (3)	10 (21)	5 (5)	22 (26)	< 0.001	0 (0)	0 (0)	0 (0)
Drugs, *per month*	11 (4)	15 (33)	8 (5)	10 (15)	3 (3)	37 (4)	0.188	0 (0)	0 (0)	0 (0)
Food, *per visit*	3 (1)	4 (3)	2 (1)	2 (3)	1 (1)	7 (0)	0.477	0 (0)	0 (0)	0 (0)
Other disease(s), *per visit*	1 (0)	7 (0)	1 (1)	7 (0)	0 (0)	N/A	N/A	N/A^e^	N/A^e^	N/A^e^
Guardian, *per visit*	1 (0)	7 (0)	1 (1)	7 (0)	0 (0)	N/A	N/A	0 (0)	0 (0)	0 (0)
MDR-TB	*n =* 64		*n =* 23		*n =* 41					
Income loss, *per month*	22 (34)	205 (121)	6 (26)	176 (80)	16 (39)	224 (174)	0.367	183 (105)^c^	149 (191)^c^	205 (149)^c^
								176 (114)^d^	123 (180)^d^	183 (149)^d^
Transportation, *per visit*	27 (42)	4 (11)	10 (43)	3 (13)	17 (41)	4 (8)	0.223	1 (2)	1 (2)	1 (2)
Food-supplements, *per month*	5 (8)	22 (34)	3 (13)	30 (26)	2 (5)	19 (7)	0.200	15 (29)	15 (30)	15 (24)
Consultation, *per visit*	0 (0)	N/A	0 (0)	N/A	0 (0)	N/A	N/A	0 (0)	0 (0)	0 (0)
Drug, *per month*	0 (0)	N/A	0 (0)	N/A	0 (0)	N/A	N/A	0 (0)	0 (0)	0 (0)
Food, *per visit*	3 (5)	1 (1)	2 (9)	1 (0)	1 (2)	2 (0)	1.000	1 (1)	1 (1)	1 (1)
Other disease(s), *per visit*	0 (0)	N/A	0 (0)	N/A	0 (0)	N/A	N/A	N/A^e^	N/A^e^	N/A^e^
Guardian, *per visit*	1 (2)	7 (0)	0 (0)	N/A	1 (2)	7 (0)	N/A	0 (0)	0 (0)	0 (0)

^a^The value of perceived needs for financial support were calculated only on the basis of information provided by those who indicated that they need financial support for each specific cost item. ^b^ Actual costs except for income loss were calculated on the basis of information from all patients; ^c^ Actual costs for income loss were calculated on the basis of information from patients who experienced job loss after diagnosis; ^d^ Actual costs for income loss were calculated on the basis of information from patients who had experienced personal income loss regardless of whether or not they had experienced job loss after diagnosis; N/A, not applicable; ^e^ During the interview, no specific question was asked on actual costs for other diseases; **P-values indicate the statistical significance of differences regarding the value of perceived needs between poor and non-poor households per cost item

. Patients who reported that they required financial support were asked about the value of support they needed. As the wide interquartile ranges show, the value of financial support required varied strongly per cost item. MDR-TB patients perceived a need for a much higher value of financial support per month for their income loss than TB patients (median of USD 205 [IQR 121] vs USD 75 (IQR 112), *P* <  0.001). However, with regard to transportation costs per treatment visit and food-supplement costs per month, the values of the financial support required did not differ between the groups. Among MDR-TB patients, the values of the financial support needed did not differ between poor and non-poor households. But in the TB group, non-poor households perceived a need for a slightly higher value of financial support for income loss, transportation, and food supplements (*P* <  0.001).

The needs perceived by patients who indicated that they needed financial support were compared with the actual costs incurred by all patients. For income loss, we compared the value of perceived needs with the actual income loss suffered by (a) patients who had experienced job loss after diagnosis and (b) patients who had experienced any income loss due to TB regardless of whether or not they had experienced job loss. Among TB patients, the median value of the perceived need for financial support to cover income loss was lower than the actual costs. Conversely, among MDR-TB patients, the perceived value of financial support was higher than their actual costs. For transportation and food-supplement costs, we compared the value of perceived needs with the costs actually incurred by all patients. The values of financial support needed for these two cost items among patients who expressed the need for support were higher than the actual median costs among all patients.

### Effect of financial support on the incidence of catastrophic costs

In our simulated scenarios, we used the median values of the financial support required (see Table 3) to determine the value of cash transfers. The value of the cash transfer for transportation costs used in the simulations was USD 4 per visit. Due to differences in the number of visits per month, this suggests that the hypothetical monthly transfer for transportation costs would vary according to treatment regimen and treatment phase (intensive and continuation phase). For TB patients undergoing Category I treatment, the average number of visit was 4 visit per month during intensive phase and one visit per month during continuation phase. These resulted in the average value of the monthly cash transfer of USD 16 in the intensive phase and USD 4 in the continuation phase. For TB patients undergoing Category II treatment, with daily visit during intensive phase, the average value of the monthly cash transfer would be USD 120 in the intensive phase and USD 4 in the continuation phase. For MDR-TB patients, the average value of the monthly cash transfer for transportation would be USD 120 in both the intensive and continuation phases. The value of the cash transfer used in the simulations for food supplements was USD 22 per month, both for TB and MDR-TB patients. For income loss, the value of the cash transfer used in the simulations was USD 75 per month for TB patients and USD 205 per month for MDR-TB patients.

If TB-affected households were given support for transportation, food supplements and income loss (Scenario VIII), the incidence of catastrophic costs would be reduced by 25 percentage points, from 36 to 11% (Table 4). This scenario would have the greatest effects on reducing the incidence of catastrophic costs than any other scenario, reducing it by 17 percentage points if patients received only 60% of the potential cash transfer (Additional file 2: Supplement 1). In this scenario, total median costs for TB patients would be reduced from USD 133 (IQR 522) to USD 0 (IQR 106) (results not reported in the table). Even so, catastrophic costs would still be faced by 11% of TB-affected households (95% confidence interval [*CI*] 8–15%). Among TB patients who still faced catastrophic costs after cash transfer, the total median costs would be reduced from USD 1527 (IQR 1023) to USD 910 (IQR 662).

**Table 4:** The incidence of catastrophic costs in eight hypothetical scenarios

Simulated hypothetical scenario		Incidence of catastrophic costs	Average budget per patient for full duration of treatment, in USD
		% (95% *CI*)	Mean (95% *CI*)
TB			
I	Baseline (no cash transfer)	36 (31–42)	–
II	Transportation costs	28 (23–33)	114 (100–127)
III	Food-supplement costs	26 (21–30)	143 (141–145)
IV	Income loss^a^	26 (21–30)	110 (88–134)
V	Income loss^b^	17 (13–21)	210 (183–236)
VI	Transportation costs and income loss	17 (13–22)	224 (197–253)
VII	Food-supplement costs and income loss	16 (12–20)	253 (231–278)
VIII	Transportation, food supplement, and income loss	11 (8–15)	367 (338–398)
MDR-TB			
I	Baseline (no cash transfer)	83 (73–92)	–
II	Transportation costs	59 (47–71)	1337 (1327–1344)
III	Food-supplement costs	77 (65–87)	269 (269–269)
IV	Income loss^a^	58 (46–70)	1309 (1010–1617)
V	Income loss^b^	52 (39–65)	1617 (1338–1899)
VI	Transportation costs and income loss	28 (18–40)	2647 (2351–2958)
VII	Food-supplement costs and income loss	53 (41–66)	1578 (1279–1886)
VIII	Transportation, food supplement, and income loss	23 (13–35)	2916 (2620–3227)

^a^The hypothetical cash transfer was assumed to have been delivered to TB patients who had experienced job loss, ^b^The hypothetical cash transfer was assumed to have been delivered to TB patients who had experienced any income loss regardless of whether or not they had experienced job loss

Although having lower effects than Scenario VIII, cash-transfer modalities for two cost items (Scenarios VI and VII) would substantially reduce the incidence of catastrophic costs: between these two scenarios, there was no significant difference (Additional file 2: Supplement 2). Of the cash-transfer modalities that would provide support for one cost item, Scenario V would provide the most substantial effect. Other modalities of providing support for one cost item (Scenarios II-IV) would provide much smaller effects than Scenario VIII (*P* <  0.001). Between Scenarios II-IV, there were no significant differences.

In the MDR-TB group, cash transfers for transportation, food supplements and income loss (Scenario VIII) would reduce the incidence of catastrophic costs by 60 percentage points, from 83 to 23%. Of all the scenarios, Scenario VIII would have the greatest effect on reducing the incidence of catastrophic costs. Under Scenario VIII, median total costs for MDR-TB patients would decrease from USD 2804 (IQR 3317) to USD 0 (IQR 801). Twenty-three percent of MDR-TB-affected households would nonetheless face catastrophic costs after the transfer (95% *CI*: 13–35%). Under the same scenario, median total costs for MDR-TB patients who still faced catastrophic costs after cash transfer would be reduced from USD 5606 (IQR 4430) to USD 2613 (IQR 3442).

**Fig. 2:**
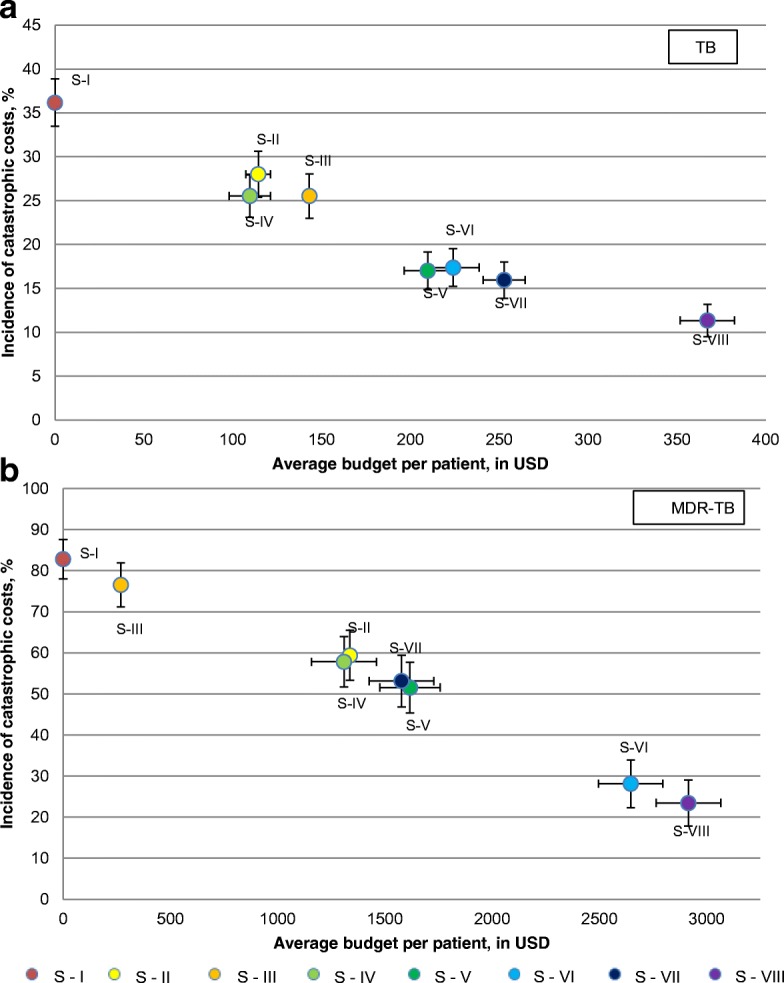
The remaining incidence of catastrophic costs and the average budget per patient for each scenario in (**a**) the TB group and (**b**) the MDR-TB group. Vertical lines in each colored circle are standard errors of the incidence of catastrophic costs. Horizontal lines in each colored circle are standard errors of the average budget per patient. MDR-TB: Multidrug-resistant tuberculosis; TB: Tuberculosis

Using the above cash-transfer values for each scenario, we estimated the average budget required per patient for the full duration of treatment under a social-protection program (Table 4, Fig. 2) Scenario VIII would produce the most significant effect, but would also require the highest average budget per patient. For the MDR-TB group, the effect and the average budget of Scenario VI were only slightly smaller than those of Scenario VIII. Other scenarios would produce a much lower effect for a much lower average budget.

**Fig. 3:**
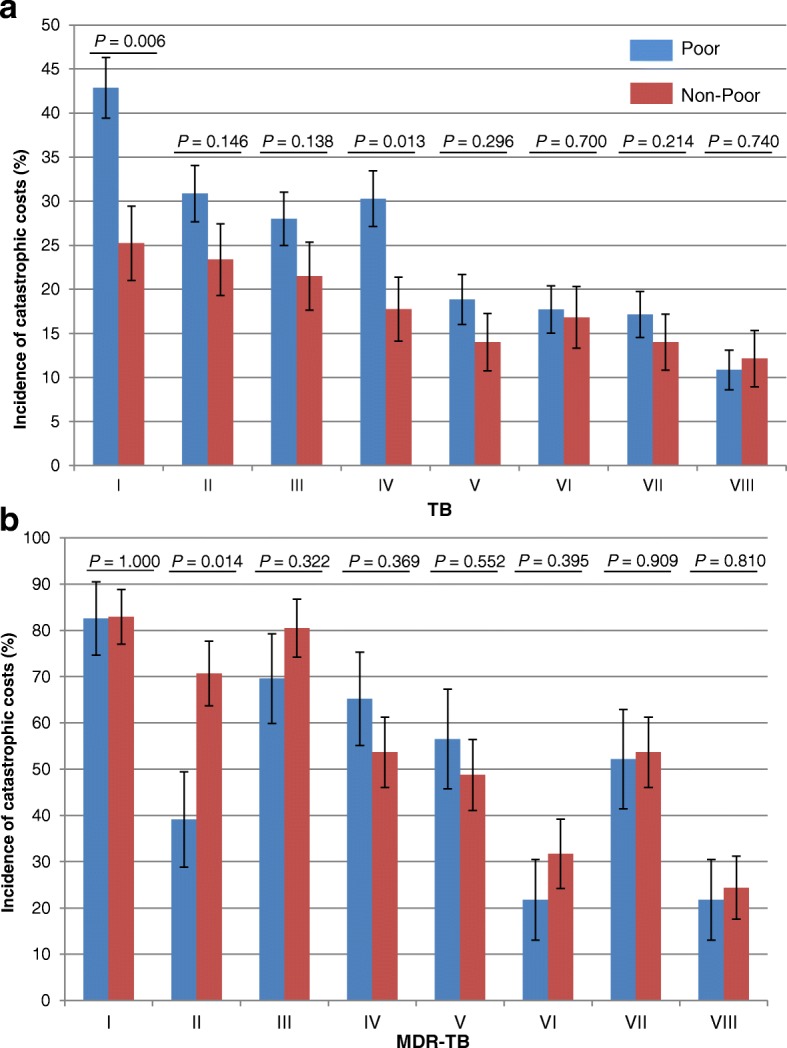
The incidence of catastrophic costs in poor and non-poor households in (**a**) TB and (**b**) MDR-TB-affected households. *P*-values indicate the differences per scenario between poor and non-poor households. MDR-TB: Multidrug-resistant tuberculosis; TB: Tuberculosis

In the TB group, cash transfers would reduce the incidence of catastrophic costs to a much greater extent in poor households than in non-poor households (Fig. 3). In Scenarios V-VIII, the gap between poor and non-poor households would disappear. A sensitivity analysis showed that the gap would also disappear under Scenarios V-VIII if patients received only 60–90% of the cash transfers (Additional file 2: Supplement 3 and Supplement 4).

In the MDR-TB group, the incidence of catastrophic costs at baseline was equally high in the poor and non-poor households. With most scenarios, the impact was similar for poor and non-poor MDR-TB households. Only cash transfers for transportation costs (Scenario II) would produce a significantly lower incidence of catastrophic costs among poor households than among non-poor ones.

## Discussion

Our results suggest that current levels of social protection in Indonesia are not enough to mitigate the socioeconomic impacts of TB, which include a high incidence of catastrophic costs, high rates of job and income loss, and a high proportion of patients who have to borrow money and sell their property. Due to these enormous impacts, TB patients urgently need social protection, mainly to cover the three costs they had indicated as most important: income loss, transportation costs, and food-supplement costs. In our simulations, the incidence of catastrophic costs were substantially reduced by a hypothetical scenario (Scenario VIII) that provided financial support for these three cost items. Nevertheless, a financial support system in which patients received fixed amounts of money for income loss, transport and food-supplement costs would not be able to reduce the incidence of catastrophic costs to 0 %, the target set by the WHO.

Our findings suggest that future policies should not rely on cash transfers for only one cost item. Although cash transfers to cover patient income loss can make a substantial contribution to reducing catastrophic costs, a single cash transfer of the sort examined in our study would not be enough to eliminate catastrophic costs. The existing types of support that patients may currently receive are equally inadequate; these mainly cover direct non-medical costs, such as food or nutritional supplement packages and travel vouchers from either government or international donors [21, 24, 25]. The impacts of financial support would be greater if cash transfers were provided for a combination of income loss, travel costs, and food-supplement costs.

Despite their substantial impact on reducing catastrophic costs, the cash transfers in our scenarios would not be enough to achieve the WHO’s target of 0 % of households facing catastrophic costs. This failure is likely to be due to the high variability of costs between patients, and particularly of the cost due to income loss, which was the greatest component of the total costs incurred due to TB. Actual monthly income losses were also higher than the cash transfers simulated in our hypothetical scenarios. For example, while the transfer was set at USD 75 per month, actual median monthly income loss among TB patients who experienced job loss was USD 75 (IQR 77) for poor patients, and USD 149 (IQR 185) for non-poor patients.

As actual costs and the perceived needs for financial support vary greatly between patients, it is difficult to determine the value of any cash transfer to be delivered. We based the value of cash transfers on the median value of patients’ perceived needs. Although the median value of cash transfer to cover transportation and food-supplement costs was higher than the median value of their actual costs, the actual value of transport and food supplements latter was sometimes higher than the cash transfer. The transfer in these cases did not cover the actual costs. However, the cash transfers simulated in our study would increase the NTP budget per capita by between approximately 46% (Scenario II) and 148% (Scenario VIII) for TB patients, and by between approximately 8% (Scenario II) and 20% (Scenario VIII) for MDR-TB patients [26]. While increasing the value of the cash transfers might be effective in terms of further reducing catastrophic costs, its affordability and sustainability should be carefully considered.

A way of reducing the incidence of catastrophic costs more effectively might be to target the financial support to those patients most likely to experience catastrophic expenditures. The targeting system could differ between settings, and could use various criteria to identify patients who need financial support [14]. Such criteria might include the determinants of catastrophic costs, such as household poverty level, job status before and after diagnosis, breadwinner status in the family, having had previous TB treatment, and experiencing adverse effects [6]. The disadvantages of such an approach would include the risks of greater stigmatization and of the greater bureaucracy needed to manage the targeting system, and may also prompt patients to pretend to remain sick in order to keep their entitlement to financial support [14, 24].

Our findings stress that the WHO’s target of eliminating the incidence of catastrophic costs requires innovations in social-protection programs. If this objective is to be attained, a combination of strategies will be required to reduce the costs patients incur in the trajectory between the pre-diagnostic phase and the end of treatment. To reduce medical costs in the pre-diagnostic and diagnostic phases, TB service delivery under the NTP – which currently provides free TB treatment in NTP-linked health services only after diagnosis – should be fully integrated into the national health insurance scheme. In turn, such integration would speed up diagnostic procedures and improve access to TB treatment, possibly reducing transport costs and potentially even income loss. However, as the proportion of costs incurred in this phase is much smaller than the proportion of costs in the treatment phase [6], the strategy would have limited impact on total cost reduction. The strategy should therefore be combined with strategies for preventing socioeconomic impacts in the treatment phase of TB.

Since income loss was the greatest cost in the treatment phase, income loss must be limited by preventing unnecessary job loss. In the formal sector, this could be done by strengthening job-security policies so as to avoid the dismissal of workers with TB and MDR-TB. In the informal sector, resolving the problem of income loss would be more of a challenge. Whatever the case, it is important to design a legal framework that provides additional social protection, not only to compensate patient’s income loss, but also to prevent further severe TB-related socioeconomic impact by ensuring that patients are covered by national health insurance.

Another possible way of reducing treatment costs is to shorten the TB treatment period [15, 27]. The development of a new TB drug regimen with a shorter treatment period is currently being evaluated [28]. Positive evidence that this shorter period is just as cost-effective would allow a reduction in direct non-medical costs and, as a result, a reduction in the likelihood of catastrophic costs [29]. For patients with MDR-TB, a possible way of reducing transportation costs and possible income loss is to increase the number of MDR-TB drug-delivery centres.

Unfortunately, the strategies for eliminating catastrophic costs named above would require considerable resources, while most of the TB high-burden countries are low- to middle-income countries with limited resources for social-protection policies [3]. If global action to combat TB does not become more innovative and is not given more funding, such countries will be left with very little chance of attaining the target stipulated in the WHO’s 2020 and 2025 milestones of 0 % of families that face catastrophic costs.

The limitations of this study fall into two main categories. First, we enrolled only TB and MDR-TB patients who had been treated in public health services, and thus not in the private sector. We did not interview patients who had dropped out of treatment, and we excluded any TB patients or suspected TB patients who had not followed standard TB diagnostic and treatment procedures. Similarly, the only MDR-TB patients we interviewed were those who had been treated in a national pulmonary referral hospital in an urban area (Jakarta). With regard to the extent of patients’ needs for social protection and to the value of cash transfers, these strict inclusion criteria may have led us to underestimate the needs of TB patients and to overestimate the needs of MDR-TB patients. MDR-TB patients treated in other MDR-TB centres or referred to PHCs after culture conversion may have lower direct non-medical costs, and may thus have lower requirements with regard to social protection.

Secondly, while these findings may apply to the island of Java, which constitutes 60% of the Indonesian population [30], they may not apply directly to the eastern part and other remote areas of Indonesia, where travel costs may be much higher than in Java, and where income loss may be much lower. It is also uncertain whether these findings will apply to other TB high-burden countries with a low- to middle-income.

## Conclusions

Indonesia’s current level of social protection is not sufficient to mitigate the socioeconomic impact of TB. Financial support for income loss, transportation costs, and food-supplement costs will substantially reduce the incidence of catastrophic costs, but financial support alone will not be sufficient to achieve the target of 0% TB-affected households facing catastrophic costs. This would require innovative social-protection policies and higher levels of domestic and external funding.

## Additional files

Additional file 1: Multilingual abstracts in the six official working languages of the United Nations. (PDF 245 kb)

Additional file 2: Supplement 1. The incidence of catastrophic costs if patients received 90%, 80%, 70% and 60% of the potential cash transfer. Supplement 2. *P*-values for the differences in catastrophic costs between scenarios. Supplement 3. The incidence of catastrophic costs between poor and non-poor if TB patients received 90, 80, 70 and 60% of the potential cash transfers. Supplement 4. The incidence of catastrophic costs between poor and non-poor if MDR-TB patients received 90, 80, 70 and 60% of the potential cash transfers. (ZIP 175 kb)

## Abbreviations

*CI*: Confidence interval
HBCs: High-burden countries
IDR: Indonesian Rupiah
IQR: Interquartile range
MDR-TB: Multidrug-resistant tuberculosis
NTP: National Tuberculosis Program
OOP: Out-of-pocket
PHC: Primary Health Centre
TB: Tuberculosis
USD: US Dollar
WHO: World Health Organization
