# Supplementary material for: Effect of financial support on reducing the incidence of catastrophic costs among tuberculosis-affected households in Indonesia: eight simulated scenarios
Source: Infect Dis Poverty. 2019 Feb 2;8:10. doi: 10.1186/s40249-019-0519-7 (PMC6359783; doi:10.1186/s40249-019-0519-7)
Supplement: Supplementary file 1 — Multilingual abstracts in the six official working languages of the United Nations. (PDF 245 kb) [file 40249_2019_519_MOESM1_ESM.pdf]

Translation of the abstract into the five official working languages of the United Nations

تأثير الدعم المادي على تقليل وقوع التكاليف الكارثية التي تُثقل كاهل الأسر المتاثرة بمرض السل في أندونيسيا: محاكاة لثمان حالات

أحمد فؤادي، تانجا إيه جيه هولينغ، مختارالدين منصور، إرلينا برهان، جان هندريك ريتشاردوس

الملخص
خلفية: تنص خطة منظمة الصحة العالمية للقضاء على مرض السل على أنه لا ينبغي لأي أسرة تتأثر بمرض السل أن تتحمل تكاليف كارثية بسبب هذا المرض. بغية إنجاز هذا الهدف، من الضروري تقديم حماية اجتماعية كافية. نظرًا لقلة الدراسات التي قامت بتقييم برامج الحماية الاجتماعية لمعرفة إن كان الهدف يُنجز بالفعل، قمنا بتقييم تأثير الدعم المادي على تقليل وقوع التكاليف الكارثية بسبب مرض السل في إندونيسيا.
المنهج: أجرينا مقابلات مع مرضى بالغين يحصلون على علاج لمرض السل أو مرض السل المقاوم لأدوية متعددة. بناءً على تقييم الاحتياجات، قمنا ببناء ثمان حالات للدعم المادي. كما قيّمنا تأثير كل من الحالات الثمان المحاكاة من خلال قياس التقليل من وقوع التكاليف الكارثية.
النتائج: قمنا بتحليل بيانات 282 مريض مصاب بالسل، و64 مريض مصاب بالسل المقاوم لأدوية متعددة. كانت نسبة وقوع تكاليف كارثية في الأسر المتأثرة بالمرضين المذكورين هي 36% و83% على التوالي. كانت احتياجات المرضى الأساسية من أجل الحصول على الحماية الاجتماعية هي الدعم المادي لتغطية المصاريف المتعلقة بالدخل المفقود، والمواصلات، والمكملات الغذائية. يعمل السيناريو المثالي حيث يتم توفير الدعم المادي للمتطلبات الثلاثة المذكورة على تقليل نسبة حدوث تكاليف كارثية في الأسر المصابة بداء السل والسل المقاوم لأدوية متعددة إلى 11% و 23% على التوالي. لكن سيتحتم على المرضى الذين يتعرضون لتكاليف كارثية في هذه الحالة دفع تكاليف متبقية عالية (متوسط 910 دولار أمريكي: نطاق انحراف رُبيعي [662]) في مجموعة السل، و 2613 دولار أمريكي [نطاق انحراف رُبيعي 3442] في مجموعة السل المقاوم لأدوية متعددة.
الاستنتاجات: ما زال وضع الحماية الاجتماعية الحالية في إندونيسيا غير كافٍ لتخفيف الآثار الاجتماعية الاقتصادية لمرض السل . على الرغم من إمكانية تقليل الدعم المادي وقوع التكاليف الكارثية من خلال تعويض الدخل المفقود، وتكاليف المواصلات، والمكملات الغذائية المجمّعة، لن يكون الدعم المادي وحده كافيًا للوصول إلى الهدف المتمثل في نسبة 0% من الأسر المتأثرة بمرض السل التي تتحمل تكاليف كارثية. يتطلب الوصول إلى الهدف المذكور استراتيجيات أكثر ابتكارًا للحماية الاجتماعية ودعمًا ماديًا أكبر محليًا وخارجيًا.

Translated from English version into Arabic by Suzan Alkhodair and Muhannad Albayk Jaam, through

TRANSLATORS
WITHOUT BORDERS

财政支持对降低印度尼西亚结核病家庭灾难性支出发生率的影响：八种模拟情景

Ahmad Fuady, Tanja A.J. Houweling, Muchtaruddin Mansyur, Erlina Burhan, Jan Hendrik Richardus

摘要：
引言：世界卫生组织的《终结结核病战略》指出,受结核病影响的家庭不应承担因结核病导致的灾难性支出。为实现这一目标，必须有充足的社会保障措施。由于很少研究评估了社会保障措施是否有利于实现这一目标，因此我们评估了财政支持对降低印度尼西亚受结核病影响家庭灾难性支出发生率的影响。

方法:我们采访了已接收治疗的成人结核病或耐多药结核病患者。基于评估需求，设计了 8 个财政支持场景。通过测量降低灾难性支出的发生率来评估每个模拟场景的效果。
结果:我们分析了 282 例结核病患者和 64 例耐多药结核病患者的资料。受影响家庭的灾难性支出发生率分别为 36%和 83%。患者的首要需求是社会保障为收入损失、运输和食品补给等相关花费给予财政支持。在最佳情景（受到以上三个方面的资助）中，患者家庭的灾难性成本发生率分别降低到 11%和 23%。但是在这种情况下，如果患者需要支付灾难性费用，则需要支付较高的其他费用(结核病组中位数为 910 美元，四分位数范围为 662；耐多药结核病组为 2613 美元，四分位数范围为 3442)。
结论:印度尼西亚目前的社会保障水平仍不足以减轻结核病的社会经济学负担。虽然对收入损失、运输成本和食品补给的综合财政支持将大大降低灾难性支出的发生率，但仅凭财政支持不足以实现受结核病影响家庭零支出灾难性成本。因此，需要更多创新性社会保障政策和更高水平的国内外部资金资助来实现这一目标。

Translated from English version into Chinese by Peng Song, edited by Jin Chen

CDC CHINESE CENTER FOR DISEASE CONTROL AND PREVENTION
NATIONAL INSTITUTE OF PARASITIC DISEASES

Effet de l’aide financière sur la réduction de l’incidence des coûts catastrophiques chez les ménages touchés par la tuberculose en Indonésie : huit scénarios de simulation

Ahmad Fuady, Tanja A.J. Houweling, Muchtaruddin Mansyur, Erlina Burhan, Jan Hendrik Richardus

Résumé
Contexte : La Stratégie de l’OMS pour mettre fin à la tuberculose stipule qu’aucun ménage touché par la tuberculose (TB) ne devrait supporter les coûts catastrophiques liés à la tuberculose. Pour que cet objectif soit atteint, il est essentiel de fournir une protection sociale adaptée. Comme seules quelques études ont évalué les programmes de protection sociale en vue de déterminer si cet objectif est atteint, nous avons évalué l'effet de l’aide financière sur la réduction de l'incidence des coûts catastrophiques liés à la tuberculose en Indonésie.
Méthodes : Nous avons interrogé des patients adultes recevant un traitement pour la tuberculose ou pour la tuberculose multirésistante (TB-MDR). Puis nous avons mis au point huit scénarios d’aide financière reposant sur l'évaluation des besoins. Nous avons évalué l'effet de chaque scénario de simulation en mesurant la réduction de l’incidence des coûts catastrophiques.
Résultats : Nous avons analysé les données recueillies sur 282 patients atteints de tuberculose et 64 patients atteints de tuberculose multirésistante. L’incidence des coûts catastrophiques sur les ménages touchés par la maladie était respectivement de 36 % et de 83 %. Les besoins principaux des patients en termes de protection sociale étaient une aide financière pour couvrir les coûts liés à la perte de revenu, au transport et aux suppléments nutritionnels. Le scénario optimal – fournissant une aide financière pour ces trois éléments – réduirait l’incidence respective des coûts catastrophiques chez les ménages touchés par la tuberculose et la tuberculose multirésistante de 11 % et de 23 %. Dans ce scénario, les patients confrontés à des coûts catastrophiques devraient toutefois payer les coûts restants (médiane de 910 dollars USD, écart interquartile [EI] 662 dans le groupe

TB, et de 2613 dollars USD, EI 3442 dans le groupe TB-MDR).
Conclusions : Le niveau actuel de protection sociale en Indonésie n’est toujours pas suffisant pour atténuer les impacts socio-économiques de la tuberculose. L’incidence des coûts catastrophiques serait considérablement réduite par une aide financière visant à combler l’ensemble des pertes (en termes de revenu et de dépenses liées au transport et aux suppléments nutritionnels), cependant, une aide financière toute seule ne suffira pas à atteindre l’objectif visant 0 % de ménages touchés par la tuberculose et faisant face à des coûts catastrophiques. Atteindre cet objectif exige des politiques de protection sociale innovantes et des niveaux plus élevés de financement national et international.

Translated from English version into French by Cendrine Strevens and Djeukeu Kana Christelle, through

Влияние финансовой поддержки на снижение тяжелого бремени расходов семей, пострадавших от туберкулеза в Индонезии: восемь разработанных сценариев.

Ахмад Фуади, Таня А.Дж. Ховелинг, Мухтаруддин Мансур, Эрлина Берхан, Ян Хендрик Рикардус

Аннотация

Общая информация: Согласно Стратегии Всемирной Организации Здравоохранения по ликвидации туберкулеза семьи, пострадавшие от туберкулеза, не должны нести огромные расходы из-за ТБ. Для достижения этой цели необходимо обеспечить соответствующую социальную поддержку. Ввиду того, что было проведено лишь немного исследований по оценке достижения целей программ социальной защиты, мы рассмотрели и оценили влияние финансовой поддержки на снижение тяжелого бремени расходов, вызванных ТБ, в Индонезии.
Методы: Мы опросили взрослых пациентов, получающих лечение по поводу ТБ или туберкулеза со множественной лекарственной устойчивостью (МЛУ-ТБ). Исходя из оценки потребностей, мы разработали восемь сценариев финансовой поддержки. Мы оценили результат каждого разработанного сценария путем оценки снижения тяжелого бремени расходов.
Результаты: Мы проанализировали данные 282 больных ТБ и 64 больных МЛУ-ТБ. Бремя соответствующих расходов в семьях больных ТБ составило 36% и 83% соответственно.

Первостепенная необходимость пациентов в социальной поддержке выражалась в покрытии расходов, связанных с потерей дохода, транспортом и пищевыми добавками. Оптимальный сценарий, при котором финансовая поддержка предоставляется по этим трем статьям, снизит тяжелое бремя расходов для семей, пострадавших от ТБ и МЛУ-ТБ, на 11% и 23% соответственно. Однако пациентам, вынужденным нести тяжелое бремя расходов по этому сценарию, придется все же нести высокие остаточные расходы (в среднем 910

долларов с интерквальным диапазоном (ИКД) 662 в группе ТБ и 2613 долларов (ИКД 3442) в группе МЛУ-ТБ).
Выводы: Нынешний уровень социальной защиты в Индонезии всё еще недостаточен для уменьшения негативного влияния ТБ на социально-экономические показатели. В то время как тяжелое бремя расходов может быть существенно уменьшено путем комбинированной финансовой поддержки потери доходов, транспортных расходов и затрат на пищевые добавки, предоставление одной лишь финансовой поддержки недостаточно, чтобы достигнуть целевых 0% расходов в семьях больных ТБ, которым приходится сталкиваться с тяжелым финансовым бременем. Достижение этой цели требует инновационной политики в области социальной защиты, а также более высокого уровня национального и внешнего финансирования.

Translated from English version into Russian by Aryuna Dorzhieva and Oksana Weiss, through

TRANSLATORS
WITHOUT BORDERS

Ocho supuestos sobre la repercusión de la ayuda económica en la reducción de la incidencia de los costos catastróficos en los hogares afectados por la tuberculosis en Indonesia

Autores: Ahmad Fuady, Tanja A.J. Houweling, Muchtaruddin Mansyur, Erlina Burhan y Jan Hendrik Richardus

Resumen

Antecedentes: La estrategia para la erradicación de la tuberculosis de la Organización Mundial de la Salud establece que ningún hogar afectado por la tuberculosis (TB) deben soportar los costos catastróficos de la enfermedad. Para lograr este objetivo, es esencial proporcionar una protección social adecuada. Dado que solo pocos estudios han evaluado los programas de protección social para determinar si este objetivo se cumple, evaluamos la repercusión de la ayuda económica en la reducción de la incidencia de los costos catastróficos de la tuberculosis en Indonesia.
Metodología: Entrevistamos a pacientes adultos en tratamiento por TB o tuberculosis resistente a múltiples fármacos (MDR). En función de las necesidades de la evaluación, desarrollamos ocho supuestos de prestación de ayuda económica. Evaluamos la repercusión de cada supuesto calculando la reducción de la incidencia de los costos catastróficos.
Resultado: Analizamos los datos de 282 pacientes de TB y 64 pacientes de MDR-TB. Los porcentajes de incidencia de los costos catastróficos fueron del 36 % y del 83 % respectivamente. La principal necesidad de protección social de los pacientes fue la ayuda económica para cubrir costos relativos a la pérdida de ingresos, transporte y suplementos nutricionales. En el supuesto óptimo, en el que se provee ayuda económica para estos tres factores, se reduciría las incidencias respectivas de los costos catastróficos en los hogares afectados por TB y MDR-TB en un 11 % y un 23 %. Sin embargo, los pacientes de este supuesto que experimentan costos catastróficos tendrían

que pagar costos remanentes altos: una mediana de 910 USD (un rango intercuartil (IQR) de 662) en el caso del grupo TB y 2613 USD (IQR de 3442) para el grupo MDR-TB.

Conclusión: El nivel actual de protección social en Indonesia sigue siendo insuficiente para mitigar el impacto socioeconómico de la tuberculosis. Si bien la incidencia de costos catastróficos podría reducirse considerablemente con ayuda económica para el total de gastos de pérdida de ingresos, transporte y suplementos nutricionales, la ayuda financiera por sí sola no será suficiente para alcanzar el objetivo del 0% de hogares afectados por la tuberculosis que enfrentan costos catastróficos. Para lograrlo, se necesitan políticas de protección social más innovadoras y una mayor financiación, tanto nacional como externa.

Translated from English version into Spanish by Marta Callava Linares and Natasha Ocampo, through

TRANSLATORS
WITHOUT BORDERS
