# Supplementary material for: Effect of financial support on reducing the incidence of catastrophic costs among tuberculosis-affected households in Indonesia: eight simulated scenarios
Source: Infect Dis Poverty. 2019 Feb 2;8:10. doi: 10.1186/s40249-019-0519-7 (PMC6359783; doi:10.1186/s40249-019-0519-7)
Supplement: Supplementary file 2 — Supplement 1. The incidence of catastrophic costs if patients received 90%, 80%, 70% and 60% of the potential cash transfer. Supplement 2. P-values for the differences in catastrophic costs between scenarios. Supplement 3. The incidence of catastrophic costs between poor and non-poor if TB patients received 90, 80, 70 and 60% of the potential cash transfers. Supplement 4. The incidence of catastrophic costs between poor and non-poor if MDR-TB patients received 90, 80, 70 and 60% of the potential cash transfers. (ZIP 175 kb) [file 40249_2019_519_MOESM2_ESM.zip › FIGURE SUPPLEMENT 3_myriad.pptx]

## Slide 1
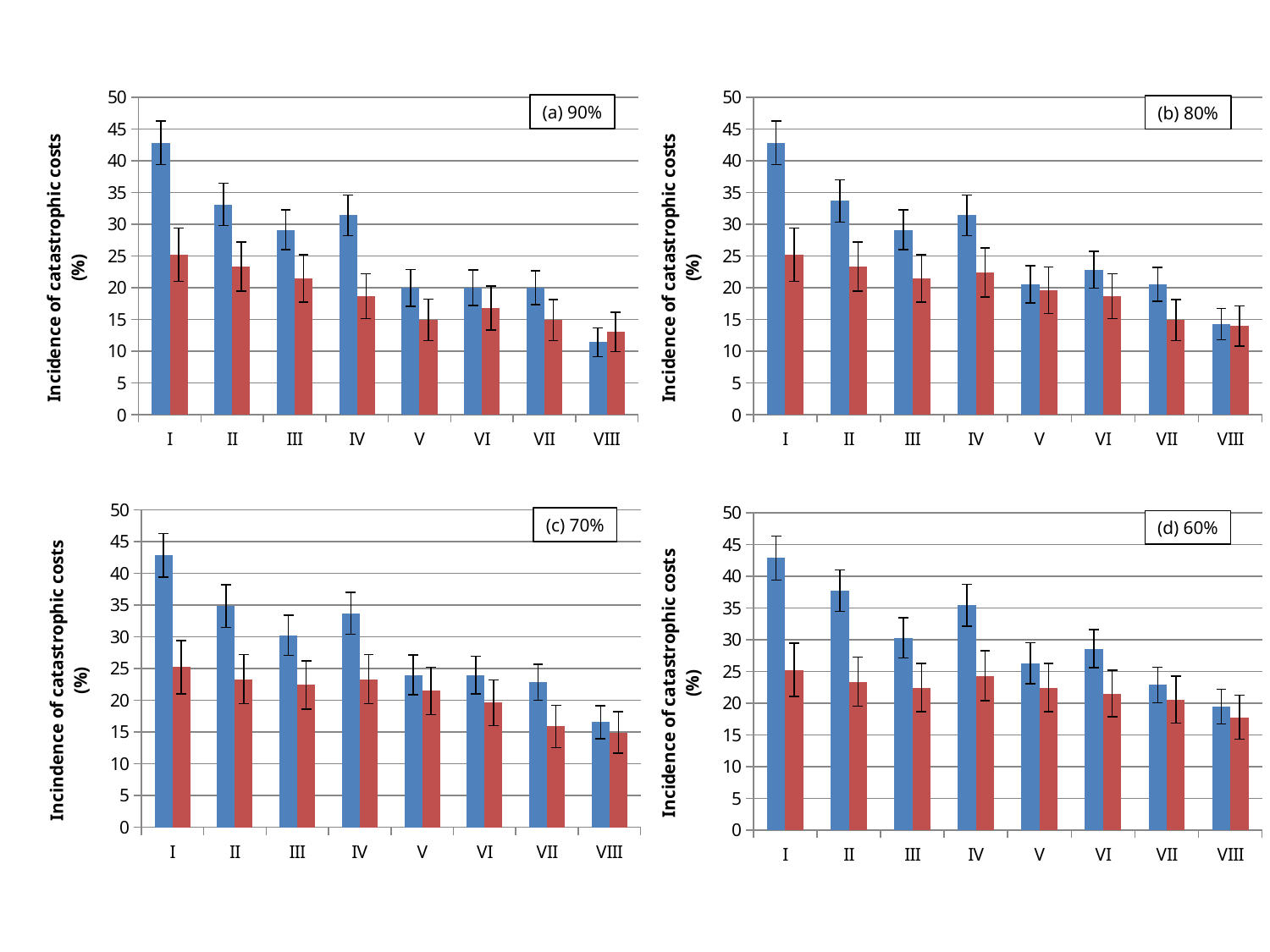

### Chart
| Category | | |
|---|---|---|
| I | 42.85714285714283 | 25.233644859813083 |
| II | 33.14285714285715 | 23.364485981308423 |
| III | 29.142857142857153 | 21.49532710280372 |
| IV | 31.428571428571427 | 18.691588785046743 |
| V | 20.0 | 14.953271028037383 |
| VI | 20.0 | 16.822429906542034 |
| VII | 20.0 | 14.953271028037383 |
| VIII | 11.428571428571418 | 13.08411214953271 |
### Chart
| Category | | |
|---|---|---|
| I | 42.85714285714283 | 25.233644859813083 |
| II | 33.714285714285715 | 23.364485981308423 |
| III | 29.142857142857153 | 21.49532710280372 |
| IV | 31.428571428571427 | 22.429906542056074 |
| V | 20.571428571428573 | 19.62616822429908 |
| VI | 22.857142857142847 | 18.691588785046743 |
| VII | 20.571428571428573 | 14.953271028037383 |
| VIII | 14.285714285714286 | 14.018691588785046 |(a) 90%
(b) 80%
### Chart
| Category | | |
|---|---|---|
| I | 42.85714285714283 | 25.233644859813083 |
| II | 34.85714285714283 | 23.364485981308423 |
| III | 30.285714285714263 | 22.429906542056074 |
| IV | 33.714285714285715 | 23.364485981308423 |
| V | 24.0 | 21.49532710280372 |
| VI | 24.0 | 19.62616822429908 |
| VII | 22.857142857142847 | 15.88785046728972 |
| VIII | 16.571428571428573 | 14.953271028037383 |
### Chart
| Category | | |
|---|---|---|
| I | 42.85714285714283 | 25.233644859813083 |
| II | 37.714285714285715 | 23.364485981308423 |
| III | 30.285714285714263 | 22.429906542056074 |
| IV | 35.42857142857146 | 24.299065420560748 |
| V | 26.285714285714263 | 22.429906542056074 |
| VI | 28.571428571428573 | 21.49532710280372 |
| VII | 22.857142857142847 | 20.56074766355139 |
| VIII | 19.428571428571427 | 17.757009345794387 |(c) 70%
(d) 60%
