# Supplementary material for: Effect of financial support on reducing the incidence of catastrophic costs among tuberculosis-affected households in Indonesia: eight simulated scenarios
Source: Infect Dis Poverty. 2019 Feb 2;8:10. doi: 10.1186/s40249-019-0519-7 (PMC6359783; doi:10.1186/s40249-019-0519-7)
Supplement: Supplementary file 2 — Supplement 1. The incidence of catastrophic costs if patients received 90%, 80%, 70% and 60% of the potential cash transfer. Supplement 2. P-values for the differences in catastrophic costs between scenarios. Supplement 3. The incidence of catastrophic costs between poor and non-poor if TB patients received 90, 80, 70 and 60% of the potential cash transfers. Supplement 4. The incidence of catastrophic costs between poor and non-poor if MDR-TB patients received 90, 80, 70 and 60% of the potential cash transfers. (ZIP 175 kb) [file 40249_2019_519_MOESM2_ESM.zip › FIGURE SUPPLEMENT 4_myriad.pptx]

## Slide 1
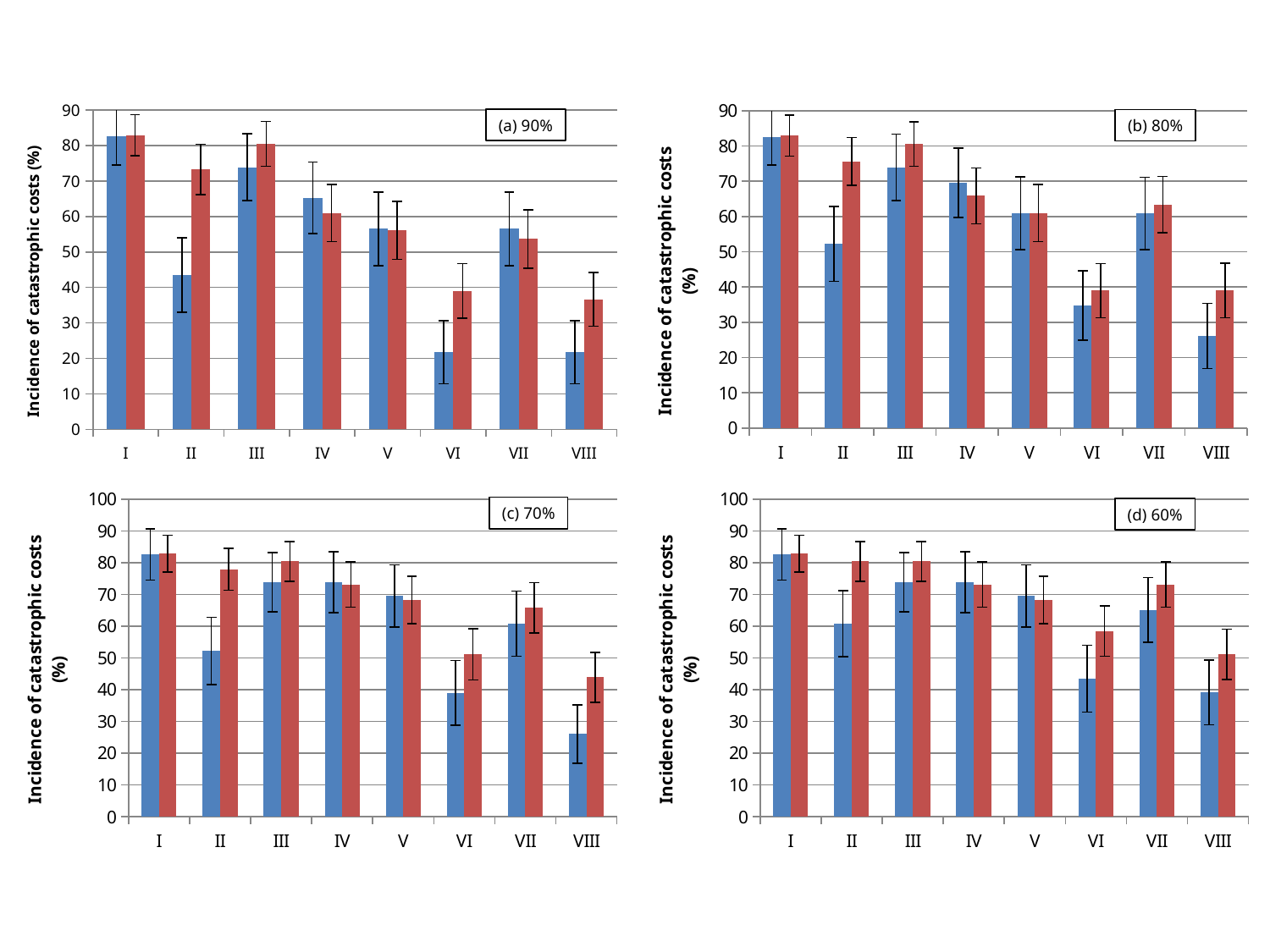

### Chart
| Category | | |
|---|---|---|
| I | 82.60869565217388 | 82.92682926829274 |
| II | 43.5 | 73.2 |
| III | 73.91304347826093 | 80.48780487804878 |
| IV | 65.21739130434787 | 60.97560975609753 |
| V | 56.52173913043481 | 56.09756097560975 |
| VI | 21.7 | 39.02439024390244 |
| VII | 56.52173913043481 | 53.65853658536585 |
| VIII | 21.73913043478261 | 36.6 |
### Chart
| Category | | |
|---|---|---|
| I | 82.60869565217388 | 82.92682926829274 |
| II | 52.2 | 75.6 |
| III | 73.91304347826093 | 80.48780487804878 |
| IV | 69.56521739130434 | 65.85365853658529 |
| V | 60.869565217391305 | 60.97560975609753 |
| VI | 34.78260869565215 | 39.0 |
| VII | 60.869565217391305 | 63.414634146341434 |
| VIII | 26.08695652173913 | 39.02439024390244 |(a) 90%
(b) 80%
### Chart
| Category | | |
|---|---|---|
| I | 82.60869565217388 | 82.92682926829274 |
| II | 52.2 | 78.0 |
| III | 73.91304347826093 | 80.48780487804878 |
| IV | 73.91304347826093 | 73.17073170731703 |
| V | 69.56521739130434 | 68.29268292682926 |
| VI | 39.1 | 51.2 |
| VII | 60.869565217391305 | 65.85365853658529 |
| VIII | 26.1 | 43.9 |
### Chart
| Category | | |
|---|---|---|
| I | 82.60869565217388 | 82.92682926829274 |
| II | 60.9 | 80.48780487804878 |
| III | 73.91304347826093 | 80.48780487804878 |
| IV | 73.91304347826093 | 73.17073170731703 |
| V | 69.56521739130434 | 68.29268292682926 |
| VI | 43.5 | 58.5 |
| VII | 65.21739130434787 | 73.17073170731703 |
| VIII | 39.130434782608695 | 51.2 |(c) 70%
(d) 60%
