# Supplementary material for: Effect of financial support on reducing the incidence of catastrophic costs among tuberculosis-affected households in Indonesia: eight simulated scenarios
Source: Infect Dis Poverty. 2019 Feb 2;8:10. doi: 10.1186/s40249-019-0519-7 (PMC6359783; doi:10.1186/s40249-019-0519-7)
Supplement: Supplementary file 2 — Supplement 1. The incidence of catastrophic costs if patients received 90%, 80%, 70% and 60% of the potential cash transfer. Supplement 2. P-values for the differences in catastrophic costs between scenarios. Supplement 3. The incidence of catastrophic costs between poor and non-poor if TB patients received 90, 80, 70 and 60% of the potential cash transfers. Supplement 4. The incidence of catastrophic costs between poor and non-poor if MDR-TB patients received 90, 80, 70 and 60% of the potential cash transfers. (ZIP 175 kb) [file 40249_2019_519_MOESM2_ESM.zip › IDOP-D-18-00286_Supplements.docx]

**Supplements**

**Supplement 1** The incidence of catastrophic costs if patients received 90%, 80%, 70% and 60% of the potential cash transfer.

| **Simulated hypothetical scenario** | | **Incidence of catastrophic costs if patients received X percent of potential cash transfer, % (95% *CI*)** | | | | |
| --- | --- | --- | --- | --- | --- | --- |
|  |  | **100%** | **90%** | **80%** | **70%** | **60%** |
| **TB** | |  |  |  |  |  |
| I | Baseline (no cash transfer) | 36 (31‒42) | - | - | - | - |
| II | Transportation costs | 28 (23‒33) | 29 (25‒35) | 30 (25‒35) | 30 (25‒36) | 32 (27‒37) |
| III | Food‒supplement costs | 26 (21‒30) | 26 (22‒31) | 26 (21‒31) | 27 (22‒32) | 27 (23‒32) |
| IV | Income loss^a^ | 26 (21‒30) | 27 (22‒31) | 28 (23‒33) | 30 (25‒35) | 31 (26‒36) |
| V | Income loss^b^ | 17 (13‒21) | 18 (14‒23) | 20 (16‒25) | 23 (18‒27) | 25 (20‒30) |
| VI | Transportation costs and income loss | 17 (13‒22) | 19 (14‒23) | 21 (17‒26) | 22 (18‒27) | 26 (22‒30) |
| VII | Food-supplement costs and income loss | 16 (12‒20) | 18 (14‒22) | 18 (14‒23) | 20 (16‒25) | 22 (17‒27) |
| VIII | Transportation, food supplement, and income loss | 11 (8‒15) | 12 (9‒15) | 14 (10‒18) | 16 (12‒20) | 19 (15‒23) |
| **MDR-TB** | |  |  |  |  |  |
| I | Baseline (no cash transfer) | 83 (73‒92) | - | - | - | - |
| II | Transportation costs | 59 (47‒71) | 63 (50‒74) | 67 (56‒78) | 69 (57‒81) | 73 (64‒84) |
| III | Food-supplement costs | 77 (65‒87) | 78 (68‒88) | 78 (68‒88) | 78 (68‒88) | 78 (68‒88) |
| IV | Income loss^a^ | 58 (46‒70) | 63 (51‒74) | 67 (55‒79) | 73 (62‒85) | 73 (63‒84) |
| V | Income loss^b^ | 52 (39‒65) | 56 (44‒68) | 61 (49‒73) | 69 (57‒81) | 69 (58‒80) |
| VI | Transportation costs and income loss | 28 (18‒40) | 33 (22‒44) | 38 (27‒50) | 47 (35‒59) | 53 (42‒66) |
| VII | Food-supplement costs and income loss | 53 (41‒66) | 55 (42‒67) | 63 (51‒75) | 64 (52‒76) | 70 (60‒81) |
| VIII | Transportation, food supplement, and income loss | 23 (13‒35) | 31 (20‒42) | 34 (23‒47) | 38 (26‒49) | 47 (36‒60) |

^a^ The hypothetical cash transfer was assumed to have been delivered to TB patients who had experienced job loss, ^b^ The hypothetical cash transfer was assumed to have been delivered to TB patients who had experienced any income loss regardless of whether or not they had experienced job loss.

**Supplement 2** *P* values for the differences in catastrophic costs between scenarios.

| **Simulated hypothetical scenario** | | **% (95% *CI*)** | ***P*-values for difference between scenarios** | | | | | | |
| --- | --- | --- | --- | --- | --- | --- | --- | --- | --- |
|  |  |  | **I** | **II** | **III** | **IV** | **V** | **VI** | **VII** |
| **TB** | |  |  |  |  |  |  |  |  |
| I | Baseline (no cash transfer) | 36 (31‒42) |  |  |  |  |  |  |  |
| II | Transportation costs | 28 (23‒33) | < 0.001 |  |  |  |  |  |  |
| III | Food-supplement costs | 26 (21‒30) | < 0.001 | 0.092 |  |  |  |  |  |
| IV | Income loss^a^ | 26 (21‒30) | < 0.001 | 0.371 | 1.000 |  |  |  |  |
| V | Income loss^b^ | 17 (13‒21) | < 0.001 | < 0.001 | 0.001 | < 0.001 |  |  |  |
| VI | Transportation costs and income loss | 17 (13‒22) | < 0.001 | < 0.001 | < 0.001 | < 0.001 | 1.000 |  |  |
| VII | Food-supplement costs and income loss | 16 (12‒20) | < 0.001 | < 0.001 | < 0.001 | < 0.001 | 0.711 | 0.454 |  |
| VIII | Transportation, food supplement, and income loss | 11 (8‒15) | < 0.001 | < 0.001 | < 0.001 | < 0.001 | 0.004 | < 0.001 | < 0.001 |
| **MDR-TB** | |  |  |  |  |  |  |  |  |
| I | Baseline (no cash transfer) | 83 (73‒92) |  |  |  |  |  |  |  |
| II | Transportation costs | 59 (47‒71) | < 0.001 |  |  |  |  |  |  |
| III | Food-supplement costs | 77 (65‒87) | 0.125 | 0.001 |  |  |  |  |  |
| IV | Income loss^a^ | 58 (46‒70) | < 0.001 | 1.000 | 0.008 |  |  |  |  |
| V | Income loss^b^ | 52 (39‒65) | < 0.001 | 0.383 | 0.001 | 0.125 |  |  |  |
| VI | Transportation costs and income loss | 28 (18‒40) | < 0.001 | < 0.001 | < 0.001 | < 0.001 | 0.001 |  |  |
| VII | Food-supplement costs and income loss | 53 (41‒66) | < 0.001 | 0.481 | < 0.001 | 0.250 | 1.000 | < 0.001 |  |
| VIII | Transportation, food supplement, and income loss | 23 (13‒35) | < 0.001 | < 0.001 | < 0.001 | < 0.001 | < 0.001 | < 0.001 | < 0.001 |

^a^ The hypothetical cash transfer was assumed to have been delivered to TB patients who had experienced job loss, ^b^ The hypothetical cash transfer was assumed to have been delivered to TB patients who had experienced any income loss regardless of whether or not they had experienced job loss.

**(Supplement - Figures)**

**Supplement 3** The incidence of catastrophic costs between poor and non-poor if TB patients received 90%, 80%, 70% and 60% of the potential cash transfers

**Supplement 4** The incidence of catastrophic costs between poor and non-poor if MDR-TB patients received 90%, 80%, 70% and 60% of the potential cash transfers
